# Evaluating the impact of empathy loss on caregiver burden in behavioral variant Frontotemporal degeneration (bvFTD)

**DOI:** 10.1002/alz.089484

**Published:** 2025-01-09

**Authors:** Lauren Fisher, Katheryn A Q Cousins, Emma Rhodes, David J Irwin, Lauren Massimo

**Affiliations:** ^1^ University of Pennsylvania, School of Nursing, Philadelphia, PA USA; ^2^ Frontotemporal Degeneration Center, University of Pennsylvania, Philadelphia, PA USA; ^3^ Penn Frontotemporal Degeneration Center, Department of Neurology, Perelman School of Medicine, University of Pennsylvania, Philadelphia, PA USA

## Abstract

**Background:**

Behavioral variant Frontotemporal Degeneration (bvFTD) is a common form of early onset dementia characterized by prominent behavioral change, including loss of empathy. Research has demonstrated that caregivers of persons living with bvFTD (PLwFTD) are more burdened, stressed, and depressed than other dementia caregivers, and that empathy loss may contribute to this high level of burden. However, it remains unclear if empathy loss contributes to caregiver burden above and beyond other neuropsychiatric symptoms. Understanding the contribution of empathy loss in the PLwFTD to caregiver burden is important for supporting caregivers. The purpose of this study is to evaluate the impact of empathy loss on caregiver burden in the context of other common neuropsychiatric symptoms.

**Methods:**

Participants included 111 caregivers to PLwFTD who were enrolled in research at the University of Pennsylvania Frontotemporal Degeneration Center and completed caregiver questionnaires that assess cognitive and emotional empathy (Interpersonal Reactivity Index [IRI]– Perspective Taking (PT) and Empathic Concern (EC) subscales, respectively), neuropsychiatric symptoms (Neuropsychiatric Inventory [NPI]), dementia severity (Clinical Dementia Rating Scale global score [CDR]), and caregiver burden (Zarit Burden Inventory [NPI]). Linear regressions were performed to evaluate the relationship between IRI subscales and caregiver burden, adjusting for dementia severity and NPI total score.

**Results:**

For PLwFTD (n = 111), mean age was 62.8 (SD = 8.74) and predominantly white, non‐Hispanic (94.6%, 99.1% respectively), and male (59.5%). Caregiver reports of lower EC (β = ‐0.80, 95% CI: ‐0.6227806 to 0.4829205, p< 0.001) and PT (β = ‐1.29, 95% CI: ‐1.8700469 to ‐0.5882883, p<0.001) were associated with increased caregiver burden. After covarying for other neuropsychiatric symptoms (NPI total score: β = 2.28, 95% CI: 1.2032696 to 3.3532612, p<.001) and dementia severity (CDR: p = 0.25), decreased PT remained significantly associated with increasing caregiver burden (β = ‐0.77, 95% CI: ‐1.4041703 to ‐0.1321910, p<0.05), but EC was no longer significant (p = 0.57).

**Conclusions:**

Empathy loss is a common behavioral symptom in PLwFTD that contributes to caregiver burden, even after accounting for other neuropsychiatric symptoms. In particular, we found that cognitive empathy or the inability of PLwFTD to understand their caregiver’s viewpoint is a major source of burden. These findings can inform interventions to support caregivers of PLwFTD.

